# *In Vitro* Investigation of the Cytotoxic Activity of Emodin 35 Derivative on Multiple Myeloma Cell Lines

**DOI:** 10.1155/2021/6682787

**Published:** 2021-01-25

**Authors:** Jing Zheng, Yingyu Chen, Zhihong Zheng, Yanxin Chen, Yujuan Chai, Wenfeng Wang, Tetsuya Asakawa, Jianda Hu

**Affiliations:** ^1^Fujian Institute of Hematology, Fujian Provincial Key Laboratory on Hematology, Fujian Medical University Union Hospital, Fuzhou, China; ^2^School of Medical Engineering, Health Science Center, Shenzhen University, Shenzhen, China; ^3^Department of Chemistry, Fuzhou University, Fuzhou 350108, China; ^4^Department of Neurosurgery, Hamamatsu University School of Medicine, Handayama, Hamamatsu, Shizuoka, Japan; ^5^Research Base of Traditional Chinese Medicine Syndrome, Fujian University of Traditional Chinese Medicine, Fuzhou 350122, China

## Abstract

**Background:**

Bortezomib is used for treating multiple myeloma (MM); however, it has considerable adverse effects. Emodin has been reported to exhibit inhibitory effects on MM cell lines. We investigated the efficacy of emodin 35 (E35), an emodin derivative, using U266 and MM1s cell lines in treating MM and the efficacy of combining bortezomib and E35.

**Methods:**

MTT assays were used to observe the effects of E35 on MM cell growth. The effects on cellular apoptosis were then observed using Annexin V/propidium iodide (PI) staining assay. The expression of apoptosis-related genes, including the caspase family, was examined. The efficacy of combining bortezomib and E35 was investigated by examining the expression of the Akt/mTOR/4EBP1 signaling pathway-related proteins.

**Results:**

We report that E35 inhibited the growth of U266 and MM1s cells by inducing cellular apoptosis. Moreover, E35 downregulated the expression of apoptosis-related genes and suppressed the phosphorylation of Akt/mTOR/4EBP1 signaling pathway-related genes, thus exhibiting synergistic effects with bortezomib. All observed effects were dose-dependent.

**Conclusion:**

The results showed that E35 exhibited cytotoxic effects in MM cell lines in protein levels. Thus, E35, particularly in combination with bortezomib, may be considered as a promising treatment for MM; however, this requires further investigation *in vivo*.

## 1. Introduction

Multiple myeloma (MM) is the second leading hematological malignancy, accounting for ∼10% of all hematological tumors [1]. Despite certain progress in therapy based on the new understanding of molecular mechanisms, MM remains generally incurable with a poor median survival. The crucial role of the NF-*κ*B signaling pathway, considered as one of the most important inflammatory pathways involved in the onset of MM [1], in the development and progression of MM has been increasingly investigated. Hence, suppressing the abnormal activation of inflammatory pathways has been an important therapeutic approach.

**Figure 1:**
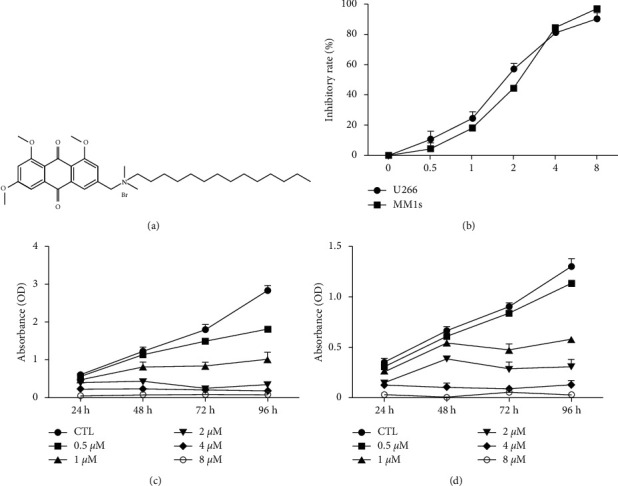
Effects of emodin derivative (E35) on myeloma cell lines. (a) Chemical structure of E35. (b) Inhibitory effects of E35 on U266 and MM1s cell growth after 48 h incubation. (c) Growth curve of U266 cells in different E35 levels. (d) Growth curve of MM1s cells in different E35 levels. Data are presented as means ± SD; OD = Optical Density.

Emodin (1,3,8-trihydroxy-6-methylanthraquinone) is a natural anthraquinone compound extracted from *Radix et Rhizoma Rhei*. Its biological activities are complex and multifaceted but include anti-inflammatory and immunomodulatory effects. Emodin has been reported to exhibit therapeutic effects on hematological tumor cell lines. A previous study reported that emodin contributes to suppressing cellular proliferation [2] and inducing apoptosis [3] in a number of leukemic cell lines by inhibiting inflammatory signaling pathways [4]. Our laboratory examined the effects of 16 types of emodin derivatives on the cell lines of hematological tumors [5, 6]. We also found that emodin induces apoptosis in resistant acute leukemia cells [7]. One emodin derivative, Emodin 35 (E35, C_34_H_50_ BrNO_5_·H_2_O; molecular weight, 631.29; Figure 1(a)), has been shown to downregulate TP53 protein expression and decrease PI3K/Akt protein phosphorylation in diffused large B cell lymphoma cells [8] while downstreaming Crk, Akt/mTOR, and MEK/ERK pathways in 32Dp210-T315I leukemia cells [9], inhibiting the cellular growth, and inducing apoptosis in leukemia cells [10]. By far, most studies focused on the effects of emodin on leukemia, and only limited studies investigated the effects of emodin on MM. In 2007, Muto et al. reported that emodin suppresses the interleukin-6-related JAK2/STAT3 pathway and causes apoptosis in MM cells by downregulating myeloid cell leukemia 1 (Mcl-1) expression [11]. In a previous study, we reported that E11, an emodin derivative, significantly inhibits proliferation and induces apoptosis of RPMI8226 and U266 MM cell lines [5].

Bortezomib has been considered as an effective strategy in treating MM [12], and the most important mechanism is the suppression of the NF-*κ*B signaling pathway [13, 14] and retention of certain tumor suppressor proteins [13]. Other mechanisms include promoting the apoptosis of MM cells [15] and inhibiting the Akt/mTOR activity [16]. However, bortezomib is associated with a number of adverse effects; the most common ones are fatigue, nausea, diarrhea, constipation, peripheral neuropathy, arthralgia, insomnia, headache, limb pain, thrombocytopenia, and upper respiratory tract infection [17–19]. Peripheral neuropathy is the most common cause of bortezomib discontinuation [20, 21]. These adverse effects may reduce the quality of life in some patients, thus restricting bortezomib use in such patients. Hence, there is an increase in the use of certain products in combination with bortezomib to achieve similar efficacy but with a reduced incidence of adverse effects.

Meanwhile, suppressing the phosphorylation of the Akt/mTOR/4EBP1 signaling pathway has been a therapeutic target in MM [22]. Many previous studies verified that emodin presents its antineoplastic effects by suppressing the Akt/mTOR pathway in hepatocytes, [23] in lung cancer cells [24], in gastric cancer cells [25], and in human breast cancer cells. [26]. Nevertheless, the role of the Akt/mTOR pathway in emodin has never been investigated. Accordingly, we hypothesize that E35 can induce apoptosis in MM cells, thus suppressing the activation and phosphorylation of the Akt/mTOR/4EBP1 signaling pathway; moreover, it may present synergistic effects with bortezomib.

In this study, we used two MM cell lines, U266 and MM1s cells, to investigate our hypothesis. We investigated the efficacy of E35 on MM cells, thus exploring its potential mechanisms via the inflammatory signaling pathway.

## 2. Methods

### 2.1. Myeloma Cell Lines

Both U266 and MM1s myeloma cell lines were obtained from the Chinese Academy of Medical Sciences, Tianjin Institute of Hematology. The cells were cultured in RPMI1640 medium (Gibco, NY, USA) with 10% fetal bovine serum (Hao Yang, China), incubated at 37°C under saturated humidity, and passaged once every 2 days. Cells that were at the logarithmic growth phase were then used for subsequent experiments.

### 2.2. Preparation of the E35 Solution

Sterile emodin (C15H10O5) was purchased from a pharmaceutical company (Qingze, China). E35, a novel emodin derivative (C34H50BrNO5H2O, Figure 1(a)), was designed and synthesized for the experiments. The purity was 98%, which was determined using high-performance liquid chromatography (HPLC). The agent was dissolved in dimethyl sulfoxide (DMSO) (Sigma, USA) in 50,000 *μ*M and stored at −20°C before use.

### 2.3. Cytotoxicity Assays

The MTT assay was used to measure the viability and proliferation of U266 and MM1s cell lines after E35 administration. U266 and MM1s cells were seeded into 96-well plates at a density of 2.0 × 10^5^ per well. The E35 dilution was subsequently added, and the final concentrations of E35 in the wells were 0.0, 0.5, 1.0, 2.0, 4.0, and 8.0 *μ*mol/L, respectively. A control group was added with the same DMSO amount as the highest concentration group. Then, 10 *μ*L of the combined MTS/PMS solution (5 mg/mL MTT) was added to each well, and the plates were incubated for 4 h. Then, the cells were incubated in a 5% CO_2_ incubator at 37°C for various durations (24, 48, 72, and 96 h). The optical density was measured with a STAT FAX-2100 spectrophotometer (Awareness Tech, USA) in 490 nm. The reference wavelength was 630 nm, and the proliferation inhibitory rate (%) and IC50 values were calculated using CalcuSyn Statistical software.

### 2.4. Annexin V/Propidium Iodide Apoptosis Assay

U266 and MM1s cells were seeded into 96-well plates, and then the E35 dilution was added. The final concentration of E35 in the wells was 1.2 *μ*mol. After incubation with E35 for 12 h, the cells were harvested and washed with PBS twice and then stained with Annexin V-FITC/propidium iodide (PI) (Becton-Dickinson, NJ, USA) as per the manufacturer's instructions. The early apoptotic cells were quantified by a BD FACSCanto II cytometer (BD Biosciences, USA).

### 2.5. Real-Time Polymerase Chain Reaction Analysis

The mRNA expression of apoptosis-related genes, namely, C-Myc, Bcl-2, Mcl-1, and Pim2, was evaluated using real-time polymerase chain reaction (RT-PCR). U266 and MM1s cells were pretreated with different concentrations of E35 or RPMI1640 for 48 h. The total mRNA was extracted and reverse-transcribed, and the transcription levels of C-Myc, Bcl-2, Mcl-1, and Pim2 were evaluated by quantitative RT-PCR using the iCycler real-time detection system (Bio-Rad, USA) in a two-step method. The hot-start enzyme was activated at 95°C for 5 min. Subsequently, cDNA was amplified for 40 cycles, which comprised denaturation at 95°C for 15 s and annealing/extension at 58°C for 30 s. A melt curve analysis was then performed (55°C for 1 min and then increased by 0.5°C every 10 s) to detect the formation of primer-derived trimers and dimers. The primer sequences were listed as follows: C-Myc: forward, 5ʹ-TCCTGGCAAAAGGTCAGAGT-3ʹ, and reverse, 5ʹ -TTGTGTGTTCGCCTCTTGA-3ʹ; Bcl-2: forward, 5ʹ -CGACGACTTCTCCCGCCGCTACCGC-3ʹ, and reverse, 5ʹ- CCGCATGCTGGGGCCGTACAGTTCC-3ʹ; Mcl-1: forward, 5ʹ-ATCTCTCGGTACCTTCGGGAGC-3ʹ, and reverse, 5ʹ-CCTG ATGCCACCTTCTAGGTCC-3ʹ; Pim2: forward, 5ʹ-CAGCCATCCAGCA CTGCCATTC-3ʹ, and reverse, 5ʹ-AGTCTGGGGAGACATGGGCTGG-3ʹ; *β*-actin: forward, 5ʹ-GGCATGGGTCAGAAGGATTCC-3ʹ, and reverse, 5ʹ-ATGTCACGCACGATTTCCCGC-3ʹ. *β*-Actin was used as the internal control. All experiments were performed in triplicate, and the data were analyzed using the 2^−ΔΔCt^ method.

### 2.6. Western Blotting Analysis

The western blotting analysis was used to measure changes in protein expression associated with treatments. The cells were exposed to E35 at varying concentrations for 48 h and then harvested and lysed. The protein concentrations were measured using the DCTM protein assay kit (Bio-Rad, USA). Electrophoresis was performed in 8%–12% sodium dodecyl sulfate polyacrylamide gel, and the samples were subsequently transferred onto a polyvinylidene fluoride membrane. The western blotting analysis was conducted as per the kit instructions. The apoptosis-related and Akt/mTOR/4EBP1 signaling pathway-related proteins were detected in U266 and MM1s cells, and the investigation with bortezomib was performed in U266 cells. The following primary antibodies were used for analysis: C-Myc, Bcl-2, Mcl-1, Pim2, poly (ADP-ribose) polymerase (PARP), caspase-3, Akt, p-Akt, mTOR, p-mTOR, 4-EBP1, p-4EBP1, EIF4E, p-EIF4E, NF-*κ*B (Danvers, USA), and *β*-actin (Fremont, USA) (internal reference). The primary antibodies were incubated overnight at 4°C. The membranes were probed with secondary antibodies (goat anti-mouse or goat anti-rabbit IgG) for 1 h at room temperature. Finally, an enhanced chemiluminescence detection system (Pierce, USA) was used for exposure. The quantitative analyses of protein expression were performed using an Image-Pro Plus system (Media Cybernetics, USA).

### 2.7. Statistical Analysis

Data were expressed as mean ± standard deviation (SD) calculated by the data from at least three independent experiments. SPSS software (v19.0.0, IBM, IL, USA) was used for statistical analysis. Data normality was analyzed using the Kolmogorov-Smirnov test. One-way analysis of variance followed by a Dunnett's post hoc test was used for multiple comparisons. We selected *p* < 0.05 as a level of significance.

## 3. Results

### 3.1. Effects of Emodin Derivative (E35) on Myeloma Cell Lines

The MTT assay results show that E35 strongly inhibited the U266 and MM1s cells based on concentration (Figure 1(b)) and time (Figures 1(c) and 1(d)) independently. The IC50 at 48 h in U266 and MM1s was 1.82 ± 0.07 (Figure 1(c)) and 2.01 ± 0.10 *μ*mol/L (Figure 1(d)), respectively.

### 3.2. E35 Induced Cell Apoptosis in U266 and MM1s Cells

**Figure 2:**
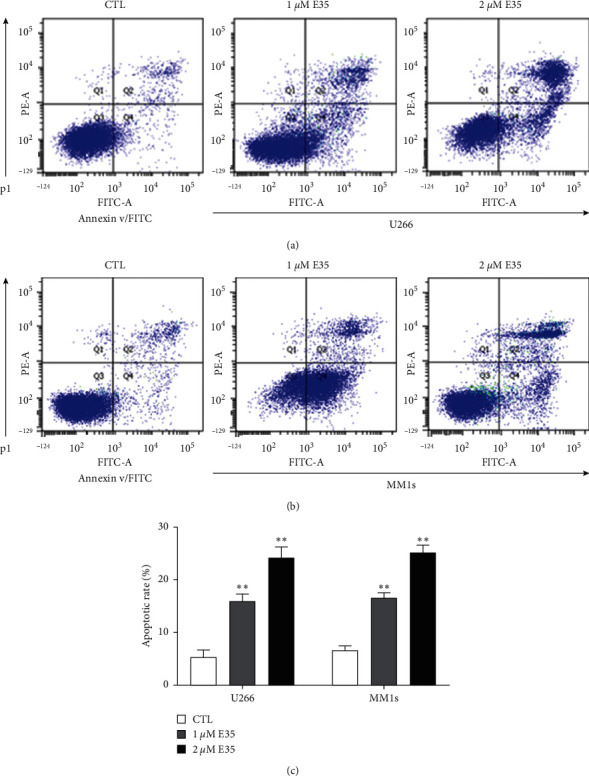
Results of the Annexin V/PI staining assay. (a) Representative images of the Annexin V/PI staining assay of E35 effects on U266 cells. (b) Representative images of the Annexin V/PI staining assay of E35 effects on MM1s cells. (c) Quantitative analysis of the Annexin V/PI staining assay of the apoptotic rate in the U266 and MM1s cell lines induced by E35. Data are presented as means ± SD. ∗∗ means *p* < 0.01 vs. control group.

Figure 2 shows the results of the Annexin V/PI staining assay. After 12 h exposure of E35, cellular apoptosis was observed in U266 (Figure 2(a)) and MM1s cells (Figure 2(b)). The apoptotic rate in control was 5.37% ± 1.53% in U266 and 6.60% ± 0.92% in MM1s. The exposure of E35 (1 *μ*M) was 15.7% ± 1.65% in U266 and 16.47% ± 1.14% in MM1s, whereas the exposure of E35 (2 *μ*M) was 24.10% ± 2.21% in U266 and 25.10% ± 1.48%% in MM1s (Figure 2(c)). Thus, the exposure of E35 significantly enhanced the apoptotic rate in both MM cell lines, and then, the dose-effect relationship was presented.

### 3.3. mRNA Expression of Apoptosis-Related Genes Affected by E35 Exposure

**Figure 3:**
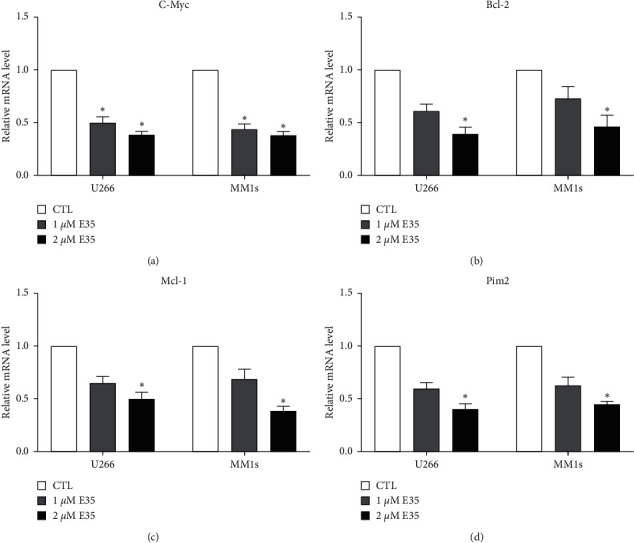
Quantitative analysis of mRNA expression of C-Myc (a), Bcl-2 (b), Mcl-1 (c), and Pim2 (d) in U266 and MM1s cell lines treated by E35. Data are presented as means ± SD. ∗ means *p* < 0.05 vs. control group.

Figure 3 shows the mRNA expression of apoptosis-related genes affected by E35. U266 and MM1s exhibited the same tendency, and the exposure of E35 for 48 h downregulated the mRNA expression of apoptosis-related genes. A high dose (2 *μ*M) induced significant downregulation (*p* < 0.05) in all genes, while a low dose (1 *μ*M) only significantly downregulated the C-Myc expression (Figure 3(a)).

### 3.4. Protein Expression of Apoptosis-Related Proteins Affected by E35 Exposure

**Figure 4:**
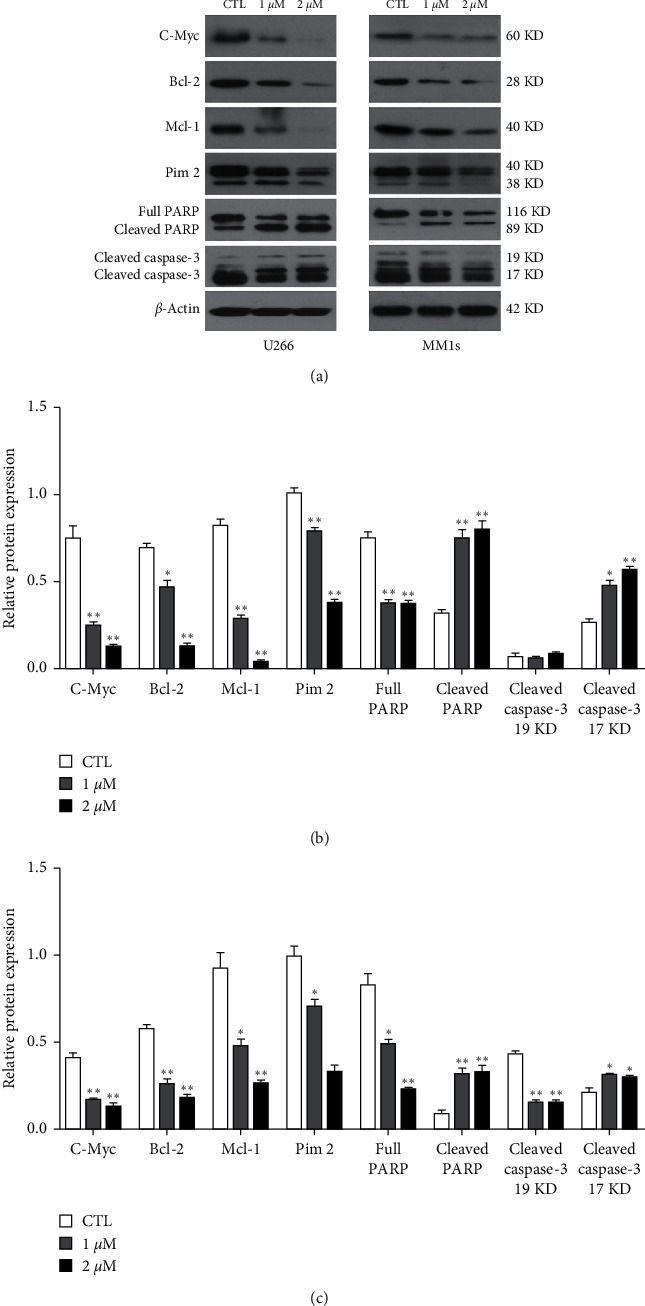
Relative expression of apoptosis-related proteins and caspase family induced by E35. (a) Representative images of western blot. (b) Quantitative results of relative expression of apoptosis-related proteins and caspase family in U266 cells induced by E35 treatment. (c) Quantitative results of relative expression of apoptosis-related proteins and caspase family in MM1s cells induced by E35 treatment. Data are presented as means ± SD. ∗ means *p* < 0.05; ∗∗ means *p* < 0.01 vs. control group.

Figure 4 shows the expression of apoptosis-related proteins, including the caspase family affected by E35 exposure. U266 and MM1s cells exhibited the same tendency. Dose-effect relationship was presented, and a higher dose was associated with stronger effects. The exposure in low (1 *μ*M) and high (2 *μ*M) doses of E35 significantly downregulated the expression of C-Myc, Bcl-2, Mcl-1, and Pim2.

With respect to the caspase family, E35 exposure significantly downregulated the expression of full PARP and significantly upregulated the expression of cleaved PARP and cleaved caspase-3 (17 kDa). As for the cleaved caspase-3 (19 kDa), E35 in both doses exhibited a significant downregulatory effect only in the MM1s cells, and no significant effect was reported in the U266 cells.

### 3.5. Expression of the Akt/mTOR/4EBP1 Signaling Pathway-Related Proteins Affected by E35 Exposure

**Figure 5:**
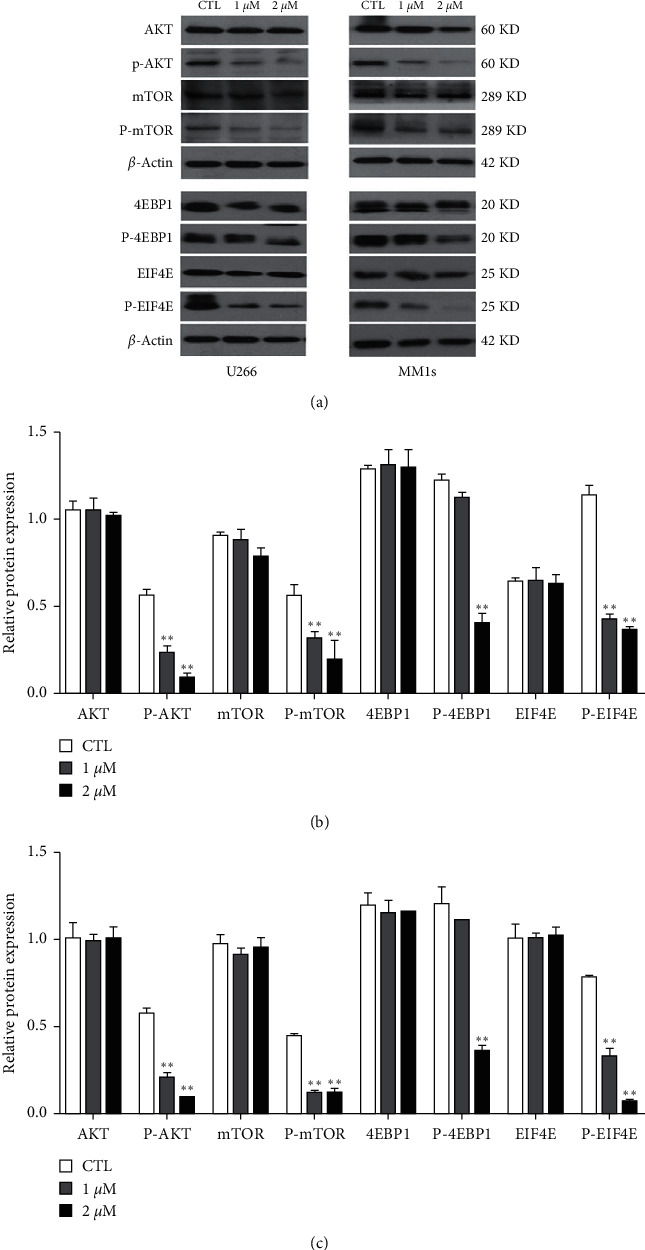
Relative expression of the Akt/mTOR/4EBP1 signaling pathway-related proteins induced by E35. (a) Representative images of western blot. (b) Quantitative results of relative expression of Akt/mTOR/4EBP1 signaling pathway-related proteins in U266 cells induced by E35 treatment. (c) Quantitative results of relative expression of Akt/mTOR/4EBP1 signaling pathway-related proteins in MM1s cells induced by E35 treatment. Data are presented as means ± SD. ∗ means *p* < 0.05; ∗∗ means *p* < 0.01 vs. control group.

**Figure 6:**
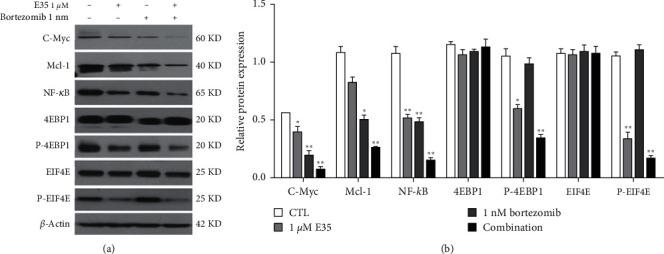
Relative expression of C-Myc, Mcl-1, NF-*κ*B, 4EBP1, p-4EBP1, EIF4E, and p-EIF4E proteins affected by administration of E35 and bortezomib in U266 cells. (a) Representative images of western blot. (b) Quantitative results of relative expression of C-Myc, Mcl-1, NF-*κ*B, 4EBP1, p-4EBP1, EIF4E, and p-EIF4E proteins. Data are presented as means ± SD. ∗ means *p* < 0.05; ∗∗ means *p* < 0.01 vs. control group.

Figure 5 shows the expression of Akt/mTOR/4EBP1 signaling pathway-related proteins affected by E35 exposure. Exposure in low (1 *μ*M) and high (2 *μ*M) doses of E35 significantly downregulated the expression of p-Akt, p-mTOR, and p-EIF4E. The expression of Akt, mTOR, 4EBP1, and EIF4E was unchanged by E35 in both doses. p-4EBP1 was only downregulated by E35 in high dose (2 *μ*M) in both U266 and MM1s cells.  Figure 6 shows that E35 administration markedly abrogated the phosphorylation of these Akt/mTOR/4EBP1 signaling pathway-related proteins. Moreover, these effects were confirmed by the administration of bortezomib, a proteasome inhibitor (Figure 6). Bortezomib, E35, and bortezomib and E35 combination significantly downregulated the protein expression of C-Myc, NF-*κ*B, p-4EBP1, and p-EIF4E. Mcl-1 expression was significantly downregulated by bortezomib and bortezomib and E35 combination. The expression of 4EBP1 and EIF4E was unchanged by E35 and bortezomib (Figure 6). The combination of E35 and bortezomib exhibited a stronger effect, thus confirming its synergistic effects.

## 4. Discussion

In this study, the cytotoxic activities of E35 on MM cells were investigated using U266 and MM1s cell lines. We report that E35 inhibited the growth of U266 and MM1s cells and induced cellular apoptosis by downregulating the expression of apoptosis-related genes (C-Myc, Bcl-2, Mcl-1, and Pim2). Caspase-3 expression (17 kDa subunit) was significantly upregulated, the full PARP was significantly downregulated, and the cleaved PARP was upregulated by E35 treatment. Moreover, we reported that E35 downregulated the phosphorylation of the Akt/mTOR/4EBP1 signaling pathway-related proteins. The downregulation and suppression of phosphorylation exhibited synergistic effects with bortezomib, a positive control. To our knowledge, this is the first study to report on the effects and underlying mechanisms of E35 on MM cells in protein levels. These results present *in vitro* evidence for the value of E35 (as well as its combination with bortezomib) as a potential therapy for MM, which requires further investigation.

The results of the MTT assay show that E35 plays an inhibitory role in the growth of U266 and MM1s cells. Moreover, such an inhibitory effect increased with an increase in concentration and treatment duration (Figures 1(b)–1(d)), indicating a dose- and time-dependent capacity of E35. The results of the Annexin V/PI staining assay indicated that E35 induced the apoptosis of MM cells (Figure 2) and downregulated mRNA/protein expression of apoptosis-related genes, including C-Myc, Bcl-2, Mcl-1, and Pim2 (Figures 3 and 4). These results were consistent with previous studies on the effects of E35 on non-Hodgkin's Lymphoma cells [8] and E11 on MM cells [5]. C-Myc and Bcl-2 play a key role in regulating apoptosis and the growth of malignancy [27–29]. Mcl-1 shares a homology domain with Bcl-2 and exerts an antiapoptotic effect [30]; Pim kinases that belong to a serine/tyrosine kinase family and that have three forms within Pim2 were reported to be closely correlated with the development and progression of MM [31]. Moreover, a synergistic function of Pim2 and C-Myc was reported to have an antiapoptotic effect; Pim2 is recognized as a partner gene of C-Myc during the induction and development of tumor [32, 33]. In the cancer state, such genes are remarkably upregulated. Some researchers believed that the high expression of these genes is good for proliferating cancer cells, and the suppression of their expression can be therefore considered as a promising therapeutic target [31]. Our results of positive control, bortezomib, show that these genes were significantly downregulated by bortezomib (Figure 6). Moreover, the synergistic effects of the combination of E35 and bortezomib lend credence to this (Figure 6). To summarize, this underscores the potential therapeutic value of E35 on MM cells.

The role of the caspase family in cellular apoptosis has been established. The activation of caspase-3 and degradation of PARP trigger cellular apoptosis. We reported that the caspase-3 expression was significantly upregulated by E35, along with the full PARP being significantly downregulated. Moreover, cleaved PARP expression was significantly upregulated by E35 treatment (Figure 4). A possible explanation is that when apoptosis was triggered by E35, the activated caspase-3 was produced and subsequently sliced the other substrates of caspase-3, including PARP. The total PARP (116 kDa) was spliced by caspase-3 between Asp216 and Gly217, thus generating two fragments (85 and 31 kDa). The endonuclease, which is negatively regulated by PARP, was then activated and started degrading the DNA in the nucleosome, thus ultimately inducing apoptosis [34]. Interestingly, we reported that the 19 kDa subunit of caspase-3 was not increased but reduced. The cause of this phenomenon is unknown. However, these data suggested a crucial but complicated role of the caspase family in E35-mediated apoptosis in MM cells.

Another key result of this study is that E35 suppressed the phosphorylation of the Akt/mTOR/4EBP1 signaling pathway-related proteins. The results of the western blot test showed that the expression of p-Akt, p-mTOR, p-EIF4E, and p-4EBP1 was significantly downregulated by E35 (p-4EBP1 only in high dose). Interestingly, however, the expression of Akt, mTOR, EIF4E, and 4EBP1 was unchanged (Figure 5). These effects were replicated when bortezomib was administered. In particular, the expression of p-4EBP1 and p-EIF4E was significantly suppressed by combining E35 and bortezomib; however, the expression levels of 4EBP1 and EIF4E were unchanged (Figure 6). These results are consistent with some previous studies [16, 22].

Recently, the Akt/mTOR/4EBP1 pathway has been recognized as one of the key signal transduction pathways that suppress cellular apoptosis through phosphorylation and subsequently induce the deactivation of downstream molecules of mTOR [35, 36]. 4EBP1 is a downstream protein of mTOR, which inhibits eIF4E (the subunit of eIF4F). When 4EBP1 is in a low phosphorylation state, it inhibits the mRNA translation by tightly binding with eIF4E while reducing interaction with cap-binding protein eIF4G to form the initial complex of eIF4F [37]. eIF4F controls the expression of many proteins that are associated with cell proliferation, expansion, and apoptosis such as Mcl-1 (antiapoptotic molecule), cyclin D1 and D3 (cell-cycle regulators), and C-Myc (oncoprotein) [38, 39]. The interpretation of our results becomes clearer. Specifically, the suppression of the expression of p-4EBP1 and p-EIF4E was associated with the downregulation of downstream eIF4F, subsequently suppressing the expression of more downstream genes (such as Mcl-1 and C-Myc), leading to the suppression of cell proliferation, expansion, and induction of cellular apoptosis. The synergistic effects of the combination of E35 and bortezomib generated a stronger effect on the suppression of phosphorylation (Figure 6). To summarize, these results suggest the crucial role that the suppression of the phosphorylation of Akt/mTOR/4EBP1 signaling pathway-related proteins plays in the E35 mechanism of action. Moreover, the NF-*κ*B protein was downregulated by the simultaneous use of E35 and bortezomib, although to a stronger extent (Figure 6). These results indicate that the suppression of the NF-*κ*B signaling pathway might play a role in the mechanism of action of E35 and/or its synergistic effects.

The present study verified the cytotoxic effects of E35, as well as its synergistic effects with bortezomib in protein levels. To obtain compelling evidence of the therapeutic effects of E35, many further verifications are required, such as investigation in terms of apoptotic rate, cytotoxicity in the cellular experiments, and *in vivo* verification which is also important. These issues will be addressed in our future study.

## 5. Conclusions

This study reported that E35 in U266 and MM1s MM cell lines exerted cytotoxic effects on protein levels. E35 inhibited the growth of cells in a dose- and time-dependent manner, induced cellular apoptosis, and downregulated the expression of apoptosis-associated genes. Caspase-3 expression was significantly upregulated, along with the full PARP being significantly downregulated. The cleaved PARP was upregulated by E35 treatment, indicating a crucial but complicated role of the caspase family in E35-mediated apoptosis. Moreover, we report that E35 suppressed the phosphorylation of Akt/mTOR/4EBP1 signaling pathway-related genes, which is a key mechanism of the effects of E35. The synergistic effects of the combination of E35 and bortezomib and the dose-effect relationship supported this idea. Our results suggested that E35, particularly the combination of E35 with bortezomib, may be considered as a promising treatment for MM. However, this requires additional investigation *in vivo*.

## Data Availability

The data used to support the findings of this study are included within the article.
